# Stroke incidence and anticoagulation treatment in patients with pacemaker-detected silent atrial fibrillation

**DOI:** 10.1371/journal.pone.0203661

**Published:** 2018-09-13

**Authors:** Emma Sandgren, Cecilia Rorsman, Nils Edvardsson, Johan Engdahl

**Affiliations:** 1 Department of Medicine, Halland Hospital Varberg, Varberg, Sweden; 2 Department of Clinical Sciences, Karolinska Institutet, Danderyd’s University Hospital, Stockholm, Sweden; 3 Sahlgrenska Academy at Sahlgrenska University Hospital, Gothenburg, Sweden; Universita degli Studi di Napoli Federico II, ITALY

## Abstract

**Background:**

Silent atrial fibrillation (AF) episodes are common but the role of anticoagulation treatment is under debate.

**Methods:**

Consecutive patients with dual-chamber pacemakers for sinus node disease or AV block/bundle branch block were retrospectively enrolled and the development of silent AF, any anticoagulation and the incidence of ischaemic stroke and dementia were recorded.

**Results:**

In total 411 patients without and 267 with known AF at implant were included. During a median follow-up of 38 months, 30% (125/411) of patients without known AF at implant were diagnosed with silent AF, 62% of those had or were prescribed anticoagulation. Heart failure (p = 0.03) and age >75 years (p = 0.0002) were risk markers for incident silent AF. In patients with known AF at implant, 80% (216/267) were on anticoagulation at implant. The annual stroke incidence was 2.1% in patients with known AF at implant, as compared to 1.9% in patients who developed silent AF during follow-up, and 1.4% in patients without AF. Vascular dementia developed in 11.2% and 6.2% respectively in patients with known AF versus no AF (p = 0.048) as well as in 5.6% of those with silent AF (p = 0.09)

**Conclusion:**

The stroke risk in our study population with an incident silent AF diagnosis may have been significantly decreased by the high proportion of anticoagulation treatment. This could imply that without this treatment the stroke risk might have been high enough to justify anticoagulation. Development of vascular dementia was twice as common among patients with known AF as compared to those witht silent AF or no AF. More data is needed to inform the optimal treatment for these patients.

## Background

Atrial fibrillation (AF) is the most common sustained arrhythmia. [1] The risk of ischaemic stroke is commonly estimated through the acronym CHA_2_DS_2_-VASc (congestive heart failure, hypertension, age, diabetes, stroke, vascular disease and female sex). In individuals with AF and increased risk of embolic stroke, anticoagulation reduces the stroke risk by 60–70%. Oral anticoagulation treatment (OAC) is currently recommended to patients with AF and CHA_2_DS_2_-VASc score ≥1. [2]

Most modern dual-chamber pacemakers have specific AF detection algorithms, while others have “atrial high-rate” detection algorithms that provide an opportunity to collect information such as the number of episodes, date and time of onset of episodes, duration of episodes, and the arrhythmia burden over time. Most models can save intra-atrial electrograms (EGMs) for detected episodes, but a small number of older models do not allow the captured arrhythmia episodes to be visually adjudicated. However, good correlation between pacemaker-detected atrial high-rate episodes (AHRE) and electrocardiographically (ECG) documented AF has been reported, particularly when the episode duration is > 5 minutes. [3]

Silent episodes of AF are common in patients with implantable devices and are associated with a significantly increased risk of ischaemic stroke. [4] The stroke risk, however, seems to be lower in patients with silent AF or episodes just diagnosed as AHRE compared to patients with overt symptomatic AF. [5]

The aim of the present study was to describe the incidence of silent AF in a population of patients implanted with a dual-chamber pacemaker and to describe their anticoagulation treatment and incidence of ischaemic stroke and vascular dementia.

## Patients and methods

**Fig 1:**
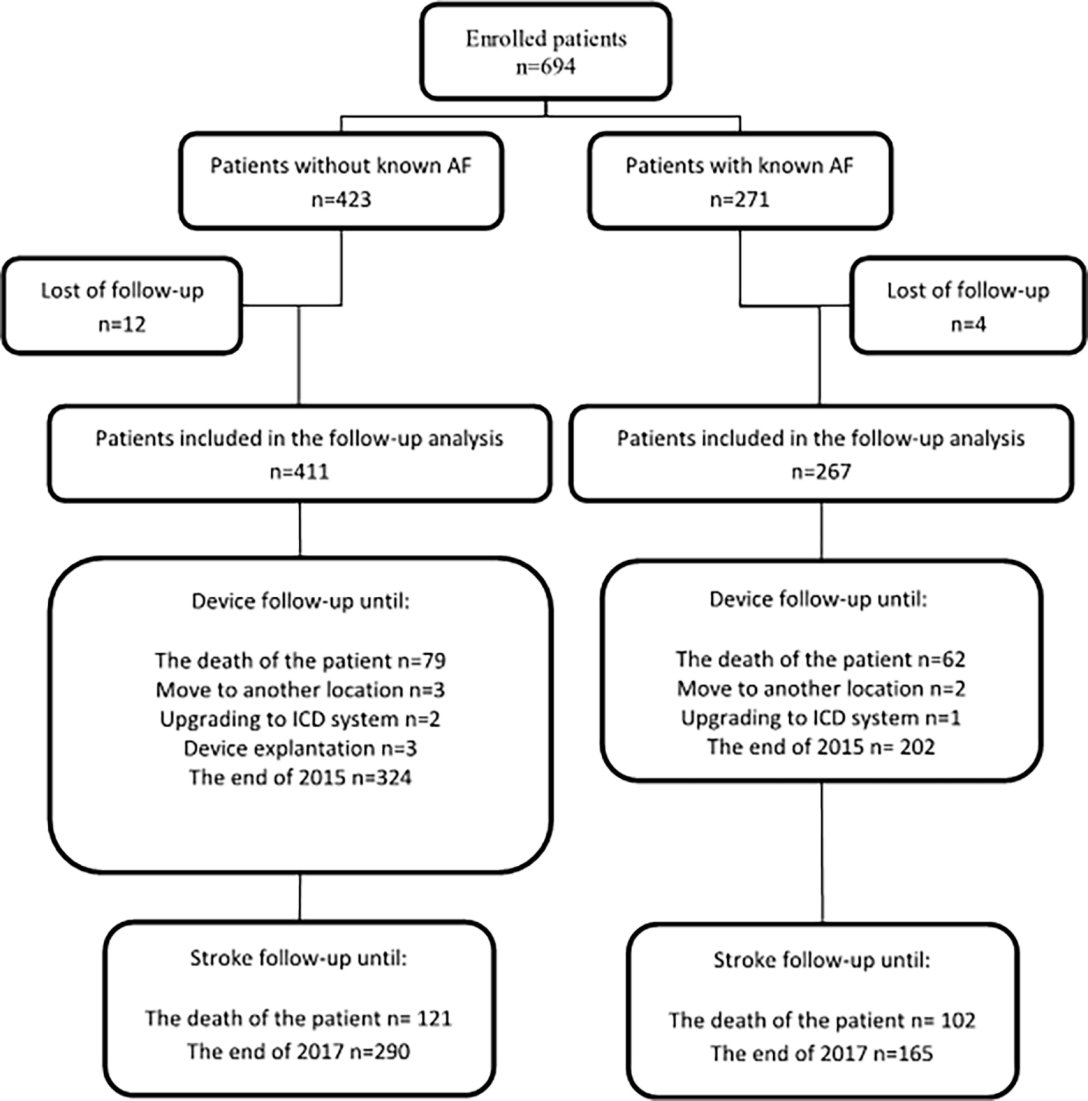
Flow chart of the study design. AF = atrial fibrillation. ICD = implantable cardioverter defibrillator.

This retrospective observational study enrolled consecutive patients implanted with a dual-chamber pacemaker (n = 687) or cardiac resynchronization therapy device (CRT-P) (n = 7), for the indication of sinus node disease (SND) or AV block (AVB)/bundle branch block, between 2010–2014 at two Swedish hospitals with a total catchment area of 300,000 inhabitants. The devices were manufactured by Medtronic (Minneapolis, Minnesota, USA, n = 588), St Jude Medical (Saint Paul, Minnesota, USA, n = 73) and Biotronik (Berlin, Germany, n = 33). For study flowchart see Fig 1.

In all patients the indication for pacemaker implantation, risk factors according to the CHA_2_DS_2_-VASc score and their anticoagulation treatment were retrieved from medical records. Clinical and arrhythmia-related data were obtained through medical records and protocols from pacemaker interrogations. Manufacturer-specific nominal settings for AHRE detection were used. The pacemaker units at the two hospitals are run by trained device nurses and biomedical technicians who independently check device diagnostics, evaluate device function and perform subsequent reprogramming as needed. Back-up cardiologists are available for adjudication of rhythm strips, assistance in programming and prescription of relevant medication. Information about ischaemic stroke and vascular dementia incidence during follow-up was retrieved from the Regional Patient Register at the end of 2017.

During follow-up the incidence of documented AF or AHRE and subsequent initiation of OAC were recorded in patients without a prior history of AF. In patients with already known AF any ongoing OAC, initiation of OAC, or any change of OAC treatment was noted. An AF diagnosis was confirmed when at least a five-minute episode was recorded by a device with an AF-detection algorithm, or by means of an electrogram rhythm strip when originally captured as an AHRE. In this study AHRE were defined as device-detected high-rate episodes not supported by rhythm strips and therefore not allowing a definite AF diagnosis to be made. Follow-up on AF detection was until the end of 2015.

The study was approved by the Regional Ethical Review Board of Lund, who found the study to be in accordance with Swedish laws and regulations for ethical approval in human research (Dnr 2015/684). The study was conducted in accordance with the principles in the Declaration of Helsinki. Patient consent was obtained through an opt-out procedure.

### Statistical analysis

Data were subjected to descriptive statistical analysis, for continuous variables via means and standard deviations (SD) and for dichotomous variables via frequencies and percentages. Univariate analysis of the continuous variables was performed using the Student’s t test and for dichotomous variables, Fisher´s exact test or chi-square test was used. Two-tailed tests were applied. A p value of <0.05 was regarded as statistically significant. A Kaplan-Meier curve was used to graphically illustrate the relationship between time from pacemaker implant and proportion of patients with silent AF. Data processing and analyses were carried out using Microsoft Excel and IBM SPSS version 24.

## Results

### Patient demographics

Of 694 enrolled patients, 395 (57%) were men. At the time of pacemaker implant, AF was already diagnosed in 271 patients while 423 patients did not have an AF diagnosis (Fig 1). Patient demographics appear in Table 1. Four patients with known AF and 12 without known AF were lost to follow-up. The others were followed for ischaemic stroke and vascular dementia incidence until death or the end of 2017. Patients with known AF at implant (n = 271) had a mean CHA_2_DS_2_-VASc score of 3.7±1.6 while those without (n = 423) had a score of 3.1±1.6 (p<0.001). Hypertension (p = 0.04), age > 75 years (p = 0.002), vascular disease (p = 0.006) and female sex (p = 0.02) were more common in patients with known AF at implant (Table 2). The CHA_2_DS_2_-VASc score was similar in patients with SND and AVB/bundle branch block, 3.3±1.6 and 3.4±1.6, respectively (p = 0.27) At the end of follow-up, 38% (102/267) of patients with known AF at implant had died as compared to 29% (121/411) of patients without known AF at implant (p = 0.02).

**Table 1:** Patient demographics at pacemaker implant separately for those with and without silent AF during follow-up.

	Silent AF (n = 125)	No AF (n = 286)	*p- value*
Age mean (years)	76.4±11	76.4±11	*n*.*s*.
< 50 years	0(0)	14(5)	***0*.*001***
50–65 years	7(6)	45(16)	
> 65 years	118(94)	227(79)	
CHA2DS2-VASc	3.3±1.6	3.3±1.6	*n*.*s*.
Congestive heart failure	12(10)	11(4)	***0*.*03***
Hypertension	80(64)	157(55)	*n*.*s*.
Diabetes	19(15)	55(19)	*n*.*s*.
History of CVI	15(12)	34(12)	*n*.*s*.
Vascular disease	38(30)	63(22)	*n*.*s*.
Sinus node disease (SND)*	29(23)	76(27)	*n*.*s*.
Atrioventricular block (AVB) I-III or bundle branch block (BBB) **	95(76)	199(70)	
SND + AVB I-III	1(0.8)	6(2.1)	
Vagal reaction	0(0)	1(0.4)	
Brady-tachy syndrome (BTS)	0(0)	4(1.4)	

* Excluding brady-tachy syndrome (BTS).

** Including bifasicular block (RBBB+LAH) + syncope and trifasicular block (RBBB+LAH+AVB I).

CVI = cerebral vascular ischemia. Values shown are mean ± SD or n(%).

**Table 2:** Distribution of the components of CHA2DS2-VASc score.

	C *n (%)*	H *n (%)*	A *n (%)*	D *n (%)*	S *n (%)*	V *n (%)*	A *n (%)*	Sc *n (%)*
All patients *n = 694*	37 (5.3)	430 (62)	433 (62)	119 (17)	92 (13)	197 (28)	188 (27)	297 (43)
AF at implant *n = 271*	13 (4.8)	186 (69)	189 (70)	43 (16)	40 (15)	93 (34)	69 (25)	136 (50)
AF not known at implant *n = 423*	24 (5.7)	244 (58)	244 (58)	76 (18)	52 (12)	104 (25)	119 (28)	161 (38)
*p- value*	*0*.*73*	***0*.*004***	***0*.*002***	*0*.*54*	*0*.*36*	***0*.*006***	*0*.*48*	***0*.*002***
Silent AF during FU *n = 125*	12 (9.6)	80 (64)	89 (71)	19 (15)	15 (12)	38 (30)	31 (25)	40 (32)
Free from AF during FU *n = 286*	11 (3.8)	157 (55)	147 (51)	55 (19)	34 (12)	63 (22)	86 (30)	115 (40)
*p- value*	***0*.*03***	*0*.*10*	***0*.*0002***	0.40	*1*.*0000*	*0*.*08*	*0*.*29*	*0*.*12*

CHA_2_DS_2_-VASc (C = congestive heart failure, H = hypertension, A_2 =_ age≥75 (2points), D = diabetes, S_2 =_ stroke/TIA/trombo-embolism (2points), V = vascular disease and Sc = female sex). AF = atrial fibrillation. FU = follow-up. Values shown are n(%).

### Silent AF diagnosed during follow-up

One hundred twenty-five out of 411 patients without known AF at implant (30%, 95%CI 26–34) had pacemaker-detected AF or AHRE during follow-up. AF was verified by stored EGM in 59 patients and by 12-lead ECG in two patients while in 64 patients the diagnosis was based on AHRE. In the latter group only one patient had a longest AHRE duration of less than one hour.

**Fig 2:**
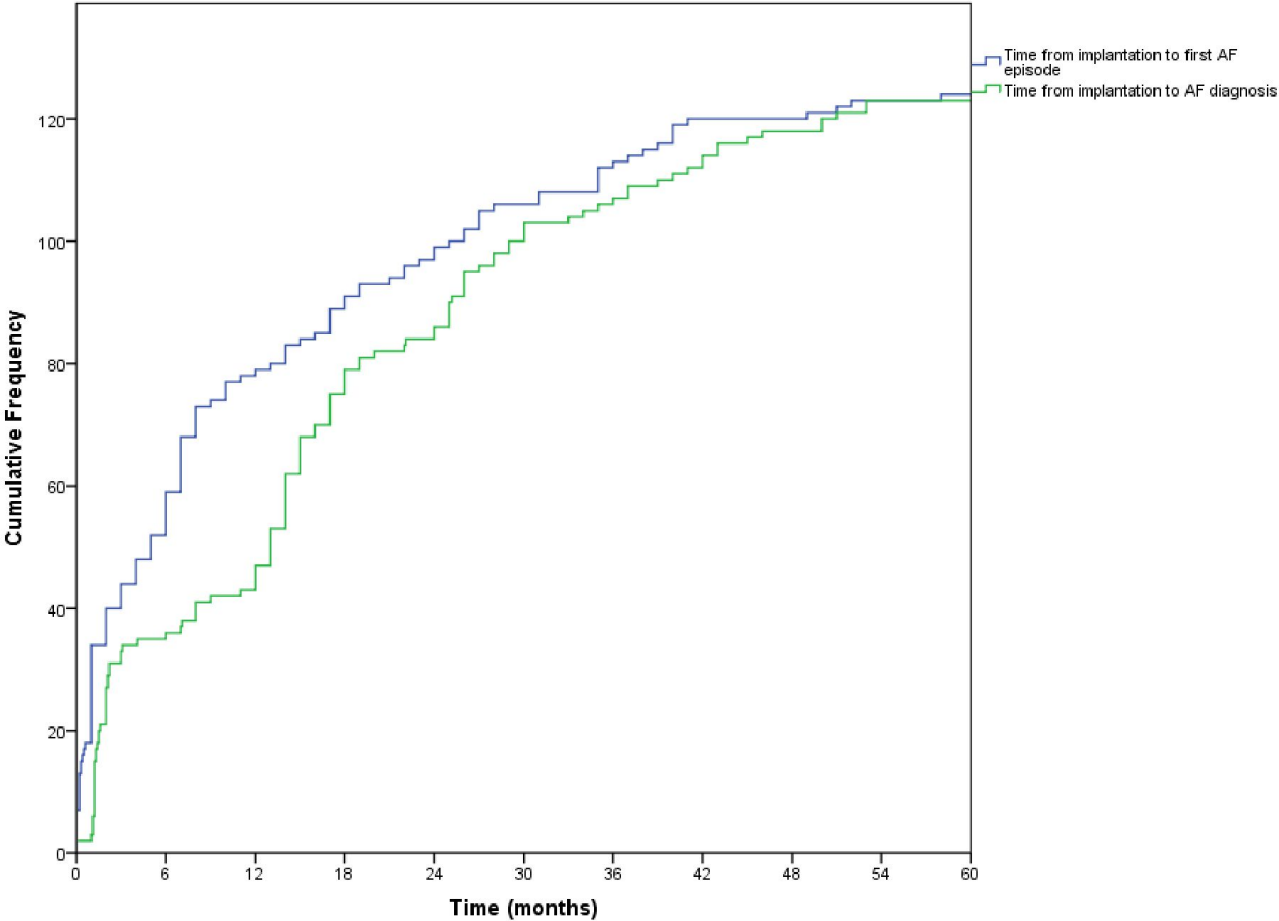
Time from pacemaker implant to silent AF episode and diagnosis. Kaplan Maier curves illustrating time from pacemaker implantation to first episode with silent AF or AHRE and time from pacemaker implantation to silent AF or AHRE diagnosis. The prominent steps in the green curve represent scheduled visits to the pacemaker unit. AF = atrial fibrillation. AHRE = atrial high-rate episode.

Two patients were diagnosed in the emergency room due to palpitations and fatigue. Among the other 123 patients who were diagnosed via the pacemaker diagnostics, two reported palpitations, three reported fatigue at the time of AF and 118 patients reported no symptoms. See Fig 2 for time to first silent AF episode and time to diagnosis.

Baseline characteristics are shown in Table 2. The median follow-up duration for patients without known AF at implantation (n = 411) was 38 months (range 2–72). The median duration from implant to the first episode of silent AF was 7 (range 0.1–62) months and from the first episode to adjudication and confirmation 5 (range 0–28) months respectively. The majority of patients, 63% (79/125), had their first episode of silent AF during the first year of monitoring, including 35% (44/125) within the first 3 months.

In patients with incident silent AF or AHRE during follow-up, the mean age at baseline, 79±11 years, was significantly higher compared to 73.3±10 years in those without (p<0.001), and their mean CHA_2_DS_2_-VASc score at implant was higher although not significantly different, 3.4±1.6 compared to 3.0±1.6 (p = 0.03). Heart failure (p = 0.03) and age >75 years (p<0.001) at baseline were predictors for the development of silent AF during.

The proportion of silent AF or AHRE respectively (p = 0.27) in patients with SND or AVB was 27% (29/109) and 32% (95/294), and the annual incidence was 8.4% and 10.2%, respectively.

### Anticoagulation treatment

Of 125 patients with silent AF, six patients already had ongoing anticoagulation treatment for reasons other than AF (warfarin n = 6). Anticoagulation treatment was prescribed in 61% (72/119) of the remaining patients.

Among patients with known AF at implantation 80% (216/271) were on anticoagulation treatment consisting of warfarin (n = 170), dabigatran (n = 24), rivaroxaban (n = 8), apixaban (n = 12) and low molecular weight heparin (n = 2).

### Stroke incidence during follow-up

In patients with known AF at implant 11% (31/267) suffered an ischaemic stroke during a mean follow-up of 67±18 months (end of 2017) with an annual incidence of 2.1%. Corresponding outcomes for patients with incident silent AF or AHRE (n = 125) and no AF (n = 286) were 10% (13/125) during mean follow-up 67±17 months, annual incidence 1.9%, and 7% (20/286) during 60±17 months of follow-up, annual incidence 1.4%, respectively (Table 3). The incidence of ischaemic stroke was significantly higher in patients with SSS compared to patients with AVB (p = 0.04).

**Table 3:** Stroke incidence during follow-up separately for patients with known AF, silent AF and no AF.

	Known AF at implant (n = 267)	AF not known at implant (n = 411)	Silent AF during FU (n = 125)	No AHRE during FU (n = 286)
Number of strokes	31	33	13	20
Toal incidence	11.0%	8.0%	10.0%	7.0%
Annual incidence	2.1%	1.6%	1.9%	1.4%
Ongoing OAC treatment	23	8	5	3

AF = atrial fibrillation. OAC = oral anticoagulation treatment. FU = follow-up.

Among patients with silent AF, 58% (72/125) had longest AF episode < 5.5h and 42% (53/125) had longest AF episode ≥ 5.5h, giving an ischaemic stroke incidence of 9.7% versus 11.3% (p = 0.78). This representing an annual incidence of 1.5% and 1.0%, respectively. For those with silent AF and ischaemic stroke during follow-up 46% (6/13) had AF episodes ≥5.5h while corresponding numbers were 37% (47/112) in those without ischaemic stroke during follow-up (p = 0.56).

## Incidence of vascular dementia during follow-up

Among patients with known AF at implant 11.2% (30/267), were diagnosed with vascular dementia during follow-up compared to 5.6% (7/125) among those with silent AF (p = 0.09) and 6.2% (18/286) for those without silent AF (p = 0.048). Almost all patients with known AF and a diagnosis of vascular dementia during follow-up 93% (28/30) were on anticoagulation treatment while 71% (5/7) of those with silent AF and a vascular dementia diagnosis had received anticoagulation treatment at the time of silent AF diagnosis.

## Discussion

This real-world observational study showed that silent AF or AHRE was common in a pacemaker population. Patients with high CHA_2_DS_2_-VASc scores were more likely to have known AF at implant or develop silent AF during follow-up. Among pacemaker patients with already known AF at implant, 80% had ongoing anticoagulation treatment, while 62% in patients without known AF either were on or received anticoagulation treatment at AF diagnosis. Nevertheless, ischaemic stroke was not uncommon and occurred with similar frequency in patients with known AF and incident AF or AHRE. Half of the patients with incident ischaemic stroke were on OAC at the time of the event. Almost one-third of patients who did not have known AF at implant were diagnosed with silent AF or AHRE during follow-up, corresponding to an annual incidence of 10%. Eleven percent of the silent AF diagnoses were made within three months, and 19% were made within the first year after implant. Almost all of them were device-detected, confirming that AF in this elderly patient population with frequent comorbidities is very often silent and not detected because of symptoms. This is possibly due to other competing symptoms or to the mostly silent nature of AF. Patients with known AF were twice as likely to receive a vascular dementia diagnosis during follow-up as patients with silent or no AF.

Once silent AF was detected, 61% of the patients, who were not already on anticoagulation treatment for other reasons received treatment based on detected silent AF. At the time when our patients were diagnosed and treated, the 2010 ESC guidelines [6] did not distinguish between symptomatic AF and silent AF and did not stress the importance of reviewing EGM when possible to verify AF. We may assume that the high percentage of patients receiving anticoagulation treatment depended on an ambition to lower the stoke risk and a belief that AF in this patient population would carry a similar risk to that in symptomatic patients. While results from the recently published ASSERT trial suggest that the stroke risk may be low and that the benefit of OAC would be in doubt [7], it is a compelling thought that the stroke risk in our study population may have been significantly decreased by the high proportion of anticoagulation treatment and that without this treatment the stroke risk might have been high enough to justify anticoagulation.

### What is an atrial high-rate episode?

An atrial high-rate episode is defined as an atrial tachyarrhythmia episode with rate > 180–190 beats/ min detected by cardiac implantable devices. [2, 8] Accordingly, besides AF, other atrial tachyarrhythmias like atrial flutter and focal atrial tachycardia (FAT) might be detected as AHRE. However, it is a minority of AHRE that are not AF [9], and to differentiate AF from atrial flutter or focal atrial tachycardia may be of less clinical importance as these arrhythmias often warrant anticoagulation treatment as well. [10]

### The pacemaker patient population, silent AF and incidence of ischaemic stroke and vascular dementia

Patients with SND and AVB are often of advanced age and have comorbidities, increasing their risk of having AF. The CHA_2_DS_2_-VASc score, assigning three points to women and two points to men over 75 years of age and with the addition of one or more comorbidities, the risk scores are frequently high and well above the threshold for anticoagulation if AF is diagnosed. This was also the case in our patients.

Silent AF was often identified early after a device implant which provided an opportunity for prompt assessment of the indication for anticoagulation treatment. There are no randomised trials demonstrating the benefit of OAC treatment in patients with silent AF [11, 12], but currently there is agreement that silent AF is associated with increased risk for stroke and systemic thromboembolism. There is a growing body of evidence establishing the link between silent AF and ischaemic stroke, and stroke risk should be managed in all patients with silent AF. [12]

The duration from the first episode to the confirmed diagnosis and assessment was often short. In the present study, 44 of 411 patients (10.1%) had their first episode with silent AF within the first three months of monitoring which is in agreement with the observations (10.1%) in the ASSERT study. [4] Thirty (68%) of these 44 patients also had their diagnoses confirmed within the first three months. However, some patients are diagnosed after several years which highlights the importance of the duration of monitoring.

Around 95% of all AHRE were not detected because of symptoms, similar to previous reports. [13, 14] A large proportion of patients had episodes with silent AF or AHRE during the first months after implantation, and the average time from when the first episode occurred to diagnosis was 4.7 months. This suggests that more frequent pacemaker interrogations during the first year after implant in particular might shorten duration from first episode to detection. Modern devices may be connected to remote monitoring which enables scheduled transmission of data as well as alerts to minimize the time from occurrence to definitive diagnosis and assessment. However, this concept was not successful for patients with implantable defibrillator (ICD) in the Impact trial [11] most likely due to low mean age, under-treatment with anticoagulation and most significantly, the study design.

We found, like Healey et. al. [15], a significant correlation between silent AF and age. In addition to age > 75 years, we found that hypertension, vascular disease and female sex were more common in patients with known AF at implant while age > 75 years and congestive heart failure were associated with a diagnosis of silent AF during follow-up. The CHA_2_DS_2_-VASc acronym is used to estimate the risk of stroke in patients with known AF but in the present study, it also identified patients with high risk of developing silent AF. [16–18]

Interestingly, considering our study size, we also found a statistically significant, higher incidence of vascular dementia in patients with known AF as compared to those with no AF, which was in accordance with Cacciatore et. al. [19] The vascular dementia incidence was similar in patients with incident AF and no AF and about half of that in patients with known AF. The lack of statistical significance of the difference in dementia incidence between patients with known AF and silent AF can probalby be explained by low numbers. The dementia incidence is notable because of the very different proportions of patients treated with OAC at baseline—80% in patients with known AF and 5% in patients with subsequent incident silent AF; during follow-up that increased in the latter group to 62%. Specifically 93% and 71% of our patients with incident dementia and known and incident silent AF, respectively, were on OAC.

In a very large register study, OAC treatment was proposed to protect against the development of dementia among AF patients. [20] In an on-treatment analysis, patients without OAC carried a risk of developing dementia of about 8% in 3 years, as compared to about 3–4% in patients with different OAC regimes. In comparison, the rate of incident dementia in our patients with known AF was 11.2% in spite of 93% of those patients being on OAC, and it was 5.6% in those with silent AF in spite of 71% of them being on OAC. The risk of dementia in our patients without AF was 6.2% while the registry study did not have a control group without AF with which to compare. Possibly pacemaker patients represent a group at higher risk than AF patients in general, and in addition patients with already clinically detected and manifest AF are likely to have more extensive cardiovascular comorbidities. Interpretations of our results should be made with caution, but if anything, this finding would seem to be in agreement with the notion that silent AF might be less harmful than symptomatic AF and that at the time of silent AF diagnosis there would still be time to initiate OAC treatment in order to, if possible, prevent or delay dementia development. An alternative interpretation could be, that patients with known AF had already had longer time to develop dementia and that the rate of dementia would increase by time—at least in the patients with incident silent AF—to catch up with the numbers in patients with known AF.

### Actual stroke incidence

Altogether 9.4% (64/678) of patients experienced an ischaemic stroke during follow-up until end of 2017. Ischaemic stroke was more common in patients with SSS compared to AVB. The actual incidence was 11%, 10% and 7% among patients with known AF, patients with silent AF or AHRE and patients with no AF, translating to an annual incidence of 2.1%, 1.9% and 1.4% respectively. In patients with silent AF, there were no significant difference in ischaemic stroke incidence among those with longest AF episodes ≥ 5.5 h compared to those with episodes < 5.5 h. The ischaemic stroke incidence occurred despite that 80% of patients with known AF and 62% of patients with silent AF was on or received either warfarin or a NOAC at the time of diagnosis, implying that the incidence would have been higher in untreated patients. In patients with stroke, 50% (32/64) had either warfarin or a NOAC at the time of stroke. The annual ischaemic stroke incidence was low compared to previously reported incidence rates for device patients [21], but there was also a larger proportion of patients receiving anticoagulation treatment. The high proportion of patients receiving anticoagulation treatment is a probable explanation for the low incidence of ischaemic stroke in patients with known AF, despite mean CHA_2_DS_2_-VASc score 3.7. [22] Even compared to the general population [23] this study population had lower incidence rate of ischaemic stroke. Possible contributing factors could be that these patients had an established healthcare contact, which may favor assessment and treatment of stroke risk factors and the fact that they were continuously monitored for arrhythmias and the high alertness in the present study to treat patients with silent AF and stroke risk factors with anticoagulation.

### Should silent AF be actively looked for?

The indication for anticoagulation treatment in patients with silent AF is still under debate, but the fact that these patients have an increased risk for cardioembolic events and that 30% of all strokes are cryptogenic [24] speaks in favour of systematic detection of silent AF and prompt assessment of the indication for anticoagulation treatment.

The combination of AF, age, sex and comorbidities determines the need for anticoagulation. In patients without known AF, a combination of the same risk factors increases the risk of having a heretofore undetected AF or developing AF in the near future. Regular and frequent rhythm controls in such patients are likely to provide important diagnostic yields.

There is an ongoing debate about whether AF is the cause of an ischaemic stroke or just a marker of patients at risk because it has been repeatedly observed that ischaemic stroke often occurs during periods of sinus rhythm. [25, 26] Although the risk of ischaemic stroke and systemic thromboembolism seems to be higher in patients with clinically detected AF compared to patients with device-detected AF the occurrence of silent AF warrants an assessment of the indication for anticoagulation, particularly if the duration is more than 24 hours.

In the current 2016 ESC guidelines an AHRE duration of more than five minutes is suggested to justify OAC treatment, provided that AF is verified by a simultaneous EGM recording. [2] If AF cannot be documented by EGM recordings, the patient characteristics, the AHRE duration and patient preferences should be considered before initiating OAC treatment. [2, 27] Recently, a consensus document from European Heart Rhythm Association (EHRA) proposed that EGMs should be reviewed when available and that AF duration > 5.5 hours would justify anticoagulation treatment when current CHA_2_DS_2_-VASc criteria are fulfilled. A shorter AF duration could merit OAC when multiple risk factors are present, but in patients with only a five-minute episode of AHRE, observation of the AF burden is recommended before OAC is started. [8]

In the absence of recommendations based on solid evidence, the EHRA has agreed on the aforementioned consensus paper [8], but ongoing large, randomised trials such as ARTESIA (NCT01938248) and NOAH-AFNET 6 (NCT02618577) that are designed to show if silent AF carries a risk enough for anticoagulation treatment are likely to increase our knowledge. In the 2016 European Society of Cardiology (ESC) guidelines [2], the benefit of OAC in patients with silent AF is stated as unclear.

### Limitations

This is an observational study from two Swedish hospitals. Our results may not necessarily be representative of those of other hospitals and regions. Per definition all patients had a clinical indication for a permanent pacemaker; we, therefore, have no control group of patients without a pacemaker. In addition no causal relationships can be concluded. The retrospective study design poses a risk of bias caused by unmeasured confounders.

## Conclusion

At present the indication for anticoagulation for silent AF episodes is uncertain. However, the results of our study imply that the stroke risk in our study population with an incident silent AF diagnosis may have been significantly decreased by the high proportion of anticoagulation treatment and that without this treatment the stroke risk might have been high enough to justify anticoagulation. Vascular dementia commonly developed, twice as often in patients with known AF than in those without known AF at implant. In spite of that, almost all these patients in both groups were on OAC. More data from larger studies are needed to inform the optimal treatment for these patients.

## Supporting information

S1 File Data set 1. Baseline data (at implantation) for all included patients.
(XLSX)

S2 File Data set 2. Data on follow-up for patients included in the follow-up analysis.
(XLSX)
